# Supplementary material for: Conditional chemoconnectomics (cCCTomics) as a strategy for efficient and conditional targeting of chemical transmission
Source: eLife. 2024 Apr 30;12:RP91927. doi: 10.7554/eLife.91927 (PMC11060718; doi:10.7554/eLife.91927)
Supplement: Figure 3—source data 3. [file elife-91927-fig3-data3.zip › Figure 3 source data3 part 1.zip!/Figure 3-Source data 3-part1/1067-9-CRISPResso_Report_c33kf3u3.zip!/CRISPResso_on_1067_12/2b.Nucleotide_percentage_quilt_around_sgRNA_agccaggtgtcgcagacgat.pdf]

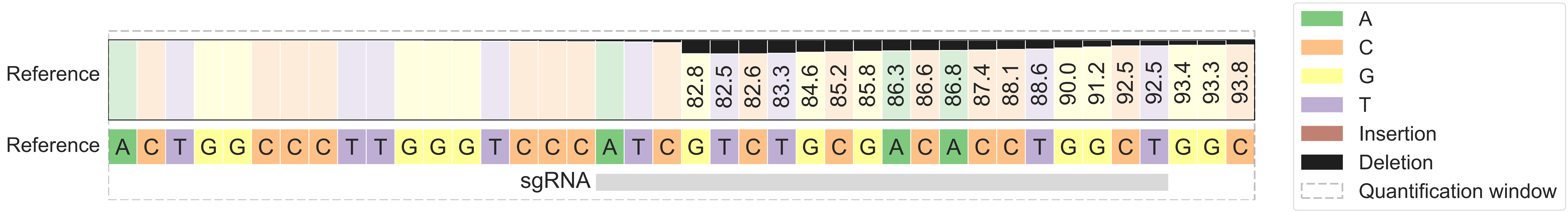

Reference
82.8 82.5 82.6 83.3 84.6 85.2 85.8 86.3 86.6 86.8 87.4 88.1 88.6 90.0 91.2 92.5 92.5 93.4 93.3 93.8
Reference
A C T G G C C C T T G G G T C C C A T C G T C T G C G A C A C C T G G C T G G C
sgRNA
A
C
G
T
Insertion
Deletion
Quantification window
